# Myasthenia Gravis Associated With Pembrolizumab for Relapsed Lung Cancer After Thymoma Resection

**DOI:** 10.7759/cureus.49767

**Published:** 2023-12-01

**Authors:** Shinya Otsuka, Kazuhiro Horiuchi, Yutaro Nagano, Noriko Kimura, Kei Hiraoka

**Affiliations:** 1 Surgery, National Hospital Organization Hakodate National Hospital, Hakodate, JPN; 2 Neurology, Hakodate Municipal Hospital, Hakodate, JPN; 3 Respiratory Medicine, Hakodate Municipal Hospital, Hakodate, JPN; 4 Diagnostic Pathology, National Hospital Organization Hakodate National Hospital, Hakodate, JPN

**Keywords:** myasthenia gravis, thymoma, immune related adverse event, pembrolizumab, immune checkpoint inhibitor

## Abstract

Immunotherapy has demonstrated clinical efficacy in patients with thymic epithelial tumors; however, there is the potential risk of serious immune-related adverse events (irAEs). Here, we report a case of myasthenia gravis (MG) associated with pembrolizumab treatment that developed after thymoma resection in a patient with lung adenocarcinoma. Symptoms of MG occurred 16 days after pembrolizumab administration and progressed rapidly, necessitating mechanical ventilation and tracheostomy. Even after tumor resection, careful monitoring is crucial for patients with thymic tumors being managed with immune checkpoint therapy, particularly regarding the development of severe irAEs.

## Introduction

The last decade has seen increased use of immune checkpoint inhibitors (ICIs) to treat various types of solid and hematological malignancies. However, ICIs pose a potential risk of immune-related adverse events (irAEs), which could be severe [1]. In particular, although the efficacy of ICIs for recurrent or refractory thymic epithelial tumors has been demonstrated, over one-third of patients with thymoma have concurrent autoimmune diseases and possibly have a higher occurrence of irAEs than those with other solid tumors [2-4].

Here, we report a rare case of myasthenia gravis (MG) crisis after the administration of pembrolizumab, an anti-programmed cell death-ligand 1 (PD-L1) inhibitor, for lung cancer relapse in a patient who underwent resection of an invasive thymoma.

## Case presentation

A 58-year-old woman without a history of autoimmune diseases presented with dyspnea for six months. Eleven years earlier, the patient underwent thoracoscopic right lower lobectomy for lung adenocarcinoma with hilar lymph node metastasis and was followed up for five years without evidence of recurrence. Adjuvant therapy was not administered.

**Figure 1:**
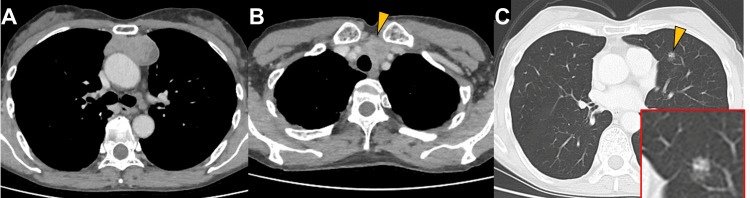
CT of the mediastinal tumor, neck mass, and lung nodule CT showing (A) a 60-mm anterior mediastinal mass in contact with the pericardium; (B) a swollen lymph node in the neck (arrowhead), and (C) multiple small nodules in bilateral lungs (arrowhead).

Chest CT revealed a 60-mm mass in the anterior mediastinum, multiple bilateral nodules in the lungs, and swollen lymph nodes in the mediastinum and neck (Figure 1).

Blood tests for anti-acetylcholine receptor (AChR) antibodies were negative, and no elevated tumor markers were observed. An anterior mediastinal tumor, lung, and lymph node metastases were suspected; tumor resection with median sternotomy was performed. The resection was incomplete owing to the involvement of the left recurrent laryngeal nerve and left phrenic nerve. A mass in the neck and nodule in the left upper lobe were also resected.

**Figure 2:**
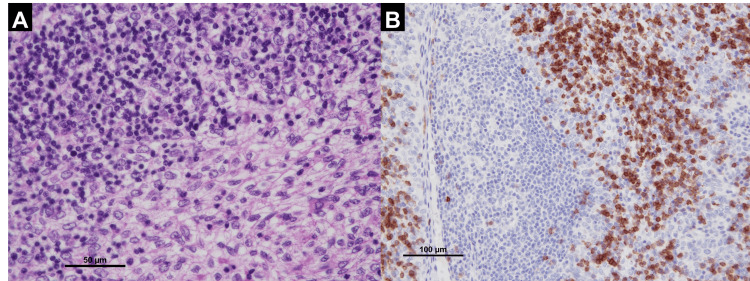
Histopathological findings of the resected thymic tumor (Type B2 thymoma) (A) Biphasic proliferation of thymocytes and lymphocytes (Hematoxylin and eosin stain. Bar: 50 μm); (B) Immature T cell (CD1a) infiltration and thymic epithelial tumor cells (CD1a stain. Bar: 100 μm).

**Figure 3:**
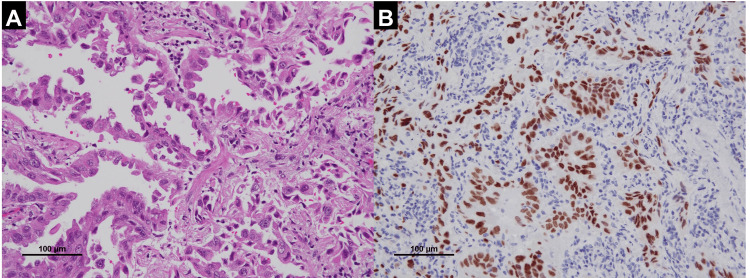
Histopathological findings of the resected left lung nodule (metastases of previous lung adenocarcinoma) (A) Papillary growth of tumor cells (Hematoxylin and eosin stain. Bar: 100 μm); (B) Tumor cells are positive for TTF-1 (TTF-1 stain. Bar: 100 μm)

On pathological examination, the anterior mediastinal tumor was diagnosed as a thymoma type B2 (Figure 2). Both the neck mass and nodule in the left lung were positive for TTF-1 on immunohistostaining and were identified as metastases of the previous lung adenocarcinoma (Figure 3).

PD-L1 expression in lung cancer was high with a tumor progression score of 100%; the tumor was negative for mutations of epidermal growth factor receptor, anaplastic lymphoma kinase, and c-ros oncogene 1. Postoperative radiation therapy for residual thymic tumors was not performed, and we chose to treat the relapsed lung cancer due to the worse prognosis associated with it. Sixteen days after initiating pembrolizumab in combination with carboplatin and pemetrexed, the patient developed hoarseness, dyspnea, and dysphagia, followed by ptosis, diplopia, and dysarthria within 24 hours. Although the patient tested negative for AChR antibodies at that time, MG was suspected. The results of the tensilon and repeated stimulation tests were positive, and MG was diagnosed.

Globulin, corticosteroids, pyridostigmine, and tacrolimus were administered intravenously. However, respiratory failure progressed owing to respiratory muscle damage, and the patient was intubated. Thirty days later, the patient underwent a tracheostomy. Plasma exchange was performed, which resulted in slow improvement, and the patient was weaned from the ventilator after three months. Steroid pulse therapy and immunoglobulin therapy were repeated; however, dysphagia persisted, and enteral nutrition was continued. One year after the development of MG, malignant pleural effusion was noted, and subsequent confirmation of a positive EGFR exon 19 deletion led to the initiation of osimertinib. However, the patient died two years and seven months after the development of MG due to the progression of lung adenocarcinoma.

## Discussion

ICIs inhibit immunosuppressive molecules expressed on immune cells and promote cytotoxic T cell responses. Antibodies targeting cytotoxic T-lymphocyte-associated protein 4 (CTLA4) - CD28 and PD-1 - PD-L1 have been used clinically. The benefits of ICIs have been demonstrated in several cancers and their application is expected to expand in the future. However, activation of the autoimmune response by ICIs and off-target effects can result in various irAEs that can be even fatal [1].

MG is one of the most clinically noteworthy irAE. It was reported that 0.12% of patients treated with nivolumab developed MG [5]. Safa et al. observed that ICI-related MG was more likely to be severe than idiopathic MG, with 45% of the patients requiring mechanical ventilation. MG often develops early (median: four weeks) after ICI initiation and patients deteriorate rapidly within a few days [6]. Although appropriate management, including corticosteroids, intravenous immunoglobulin, and plasmapheresis based on mechanical ventilation, yields a favorable prognosis, MG can still be life-threatening. One study on pembrolizumab reported a 30% mortality rate in patients who developed MG, and less than half of the patients achieved full symptom remission [7]. In that study, only 32% of patients with MG tested positive for AChR antibodies.

On the contrary, the therapeutic efficacy of ICIs has been reported for thymic epithelial tumors that have progressed following platinum-based chemotherapy and in those with distant metastases despite the risk of irAEs [4,8-10]. In a phase 2 study of pembrolizumab in 40 patients with recurrent thymic carcinoma after chemotherapy, 15% developed serious autoimmune disorders [8]. In addition, Cho et al. reported that 71% of patients with recurrent thymoma and 15% of those with thymic carcinoma who received pembrolizumab developed grade 3 or higher irAEs, including one patient with thymic carcinoma who developed grade 4 MG after two courses of pembrolizumab [9].

In the present case, the patient developed MG in the middle of the first cycle, and acute exacerbation was observed within 24 hours. Tests for anti-acetylcholine receptor antibodies were negative, even at the onset of MG. This suggests that clinical suspicion for MG and prompt treatment are crucial.

## Conclusions

Immunotherapy for patients with thymic epithelial tumors is associated with several irAEs, including MG, even if the anti-acetylcholine receptor antibody test is negative after tumor resection. Based on the risk of severe irAEs, treatment strategy including ICIs should be carefully considered.
